# Recombinant α-actinin subunit antigens of *Trichomonas vaginalis* as potential vaccine candidates in protecting against trichomoniasis

**DOI:** 10.1186/s13071-017-2009-8

**Published:** 2017-02-16

**Authors:** Yi-Ting Xie, Jiang-Mei Gao, Ya-Ping Wu, Petrus Tang, Geoff Hide, De-Hua Lai, Zhao-Rong Lun

**Affiliations:** 1 Center for Parasitic Organisms, State Key Laboratory of Biocontrol, School of Life Sciences and Key Laboratory for Tropical Disease and Control of the Ministry of Education, Zhongshan College of Medicine, Sun Yat-Sen University, Guangzhou 510275, The People’s Republic of China; 2 Bioinformatics Core Laboratory, Chang Gung University, Taoyuan, 333 Taiwan; 3 Ecosystems and Environment Research Centre and Biomedical Research Centre, School of Environment and Life Sciences, University of Salford, Salford M5 4WT, UK

**Keywords:** *Trichomonas vaginalis*, Alpha-actinin, Recombinant protein, Vaccine

## Abstract

**Background:**

Human trichomoniasis caused by *Trichomonas vaginalis* is one of the most common sexually transmitted diseases with more than 200 million cases worldwide. It has caused a series of health problems to patients. For prevention and control of infectious diseases, vaccines are usually considered as one of the most cost-efficient tools. However, until now, work on the development of *T. vaginalis* vaccines is still mainly focused on the screening of potential immunogens. Alpha-actinin characterized by high immunogenicity in *T. vaginalis* was suggested as a promising candidate. Therefore, the purpose of this study was to evaluate the protective potency of recombinant α-actinin against *T. vaginalis* infection in a mouse intraperitoneal model.

**Methods:**

Two selected coding regions of α-actinin (ACT-F, 14–469 aa and ACT-T, 462–844 aa) amplified from cDNA were cloned into pET-32a (+) expression vector and transfected into BL21 cells. After induction with IPTG and purification with electroelution, the two recombinant fusion proteins were emulsified in Freund’s adjuvant (FA) and used to immunize BALB/C mice. Following intraperitoneal inoculation with *T. vaginalis*, the survival rate of mice was monitored for the assessment of protective potency. After immunization, the antibody level in mouse serum was assessed by ELISA, splenocyte proliferation response was detected with CCK8 and cytokines in the supernatant of splenocytes were quantified with a cytometric bead-based assay.

**Results:**

We successfully obtained purified ACT-F (70.33 kDa) and ACT-T (61.7kDa). Both recombinant proteins could provide significant protection against *T. vaginalis* challenge, especially ACT-T (with 100% protection within one month). Meanwhile, high levels of specific total IgG and subtypes (IgG1 > IgG2a) were detected in sera from the immunized mice. Our results also revealed a statistically significant increase in splenocyte proliferation and related cytokine (IFN-γ, IL-6, IL-17A and IL-10) production after repeated stimulation with the corresponding antigens in vitro.

**Conclusions:**

Immunization with both ACT-F and ACT-T could confer partial to complete protection and trigger strong Th1/Th2 mixed humoral and cellular immune responses in the mouse host. This suggested that recombinant α-actinin subunit antigens may be promising vaccine candidates against trichomoniasis.

## Background

Trichomoniasis is one of the most common worldwide sexually transmitted illnesses. It is caused by *Trichomonas vaginalis* (TV) which is a facultative anaerobic flagellated parasite. It was estimated by WHO that 276 million people were infected with *T. vaginalis* globally in the age range from 15 to 49 in 2008 [1] and this had increased by 11% compared to 2005 [2]. The huge number of patients with trichomoniasis suggest that there is an urgent requirement to increase public health education and to provide proper protection.

In general, *T. vaginalis* resides in the female genital tract, causing vaginitis, urethritis and cervicitis [3]. It can also cause post-abortion infection, premature labour and complications caused by underweight offspring [4, 5]. Women infected with trichomonas are often asymptomatic, while about one-third of them may develop symptomatic infection within 6 months [6]. Men infected with *T. vaginalis* are mostly asymptomatic or show mild symptoms making them unaware of the parasite infection. Consequently, being carriers, men can transmit the parasite to their partners during sex [7]. Although *T. vaginalis* usually only causes mild symptoms in men, chronic infection still results in chronic inflammatory stimulation if left without proper treatment. Interestingly, it has been demonstrated that *T. vaginalis* infection is associated with aggressive prostate cancer [8, 9]. In addition, many reports have indicated that *T. vaginalis* infection could significantly increase the risk of transmission of human immunodeficiency viruses (HIV) [6, 10] and hepatitis viruses [11]. It has also been suggested to be associated with cervical cancer [12].

For clinical treatment of trichomoniasis, metronidazole is a widely used and effective compound. However, it may not be suitable for use in pregnant women as it is mutagenic in bacteria, carcinogenic in mice and, with the ability to cross the placenta, may result in teratogenicity in the fetus [13, 14]. Additionally, a gradual increase in the prevalence of metronidazole-resistant *T. vaginalis* strains has been reported during clinical treatment [7]. Therefore, an effective alternative to chemically synthesized compounds is urgently needed.

Besides using synthesized compounds for the treatment of *T. vaginalis* infection, vaccination is suggested as a possible effective way to protect against pathogen infection. To our knowledge, the priority to develop a vaccine against *T. vaginalis* has been understated compared with other medically important human parasitic protozoans. Pioneering research can be dated back to 1960s, where 100 women affected with refractory trichomoniasis were treated using heat-killed *T. vaginalis* administered by intravaginal inoculation [15]. The results showed that clinical symptoms in 89% of vaccinated patients showed improvement and that *T. vaginalis* in 40% of the patients was eliminated [15]. Unfortunately, to our knowledge, a similar trial has never been repeated to pursue an effective vaccine [16]. Perhaps, this was caused by the cumbersome approach such as the need to inject different doses of the inactivated *T. vaginalis* intradermally into the female cervix, fornix of the vagina and vaginal wall at 6 to 12 sites [15]. In two preliminary trials, in animal models, mice were subcutaneously immunized with whole *T. vaginalis* cells emulsified in adjuvant and the results showed some protection in a subsequent vaginal *T. vaginalis* challenge [17, 18]. In another study, intranasal immunization with a 62-kDa proteinase of *T. vaginalis* provided sufficient protection in mice [19]. However, until now, work on the development of *T. vaginalis* vaccines has been mainly focused on the screening of potential immunogens.

The published genome sequence of *T. vaginalis* will certainly provide valuable molecular information to better understand the biology of this pathogen [20]. Alpha-actinin (TVAG_190450; AF072678.1) is a predicted 115 kDa actin binding protein in *T. vaginalis*. Its function mainly involves actin cross-linking and it plays a fundamental role in cell motion and morphological changes [21]. The amoeba-like morphology is required for pathogenicity and phagocytosis, the latter being an essential procedure for *T. vaginalis* ingestion of iron, lipids, nucleotides and other nutrients [22, 23]. To date, it has been demonstrated that α-actinin is one of the most common immunogens that can be detected in the sera from women infected with *T. vaginalis* [24]. It was also reported that α-actinin was conserved across numerous *T. vaginalis* strains. Thus, when isolated from a single trichomonas strain, this imunogen could induce corresponding antibodies to different epitopes in different strains [25].

Considering that α-actinin has such an important role in *T. vaginalis* survival and high immunogenicity, it shows great potential to be used in the development of a vaccine against this parasite. Recently subunit vaccines have become a popular choice in various pathogens due to their improved avoidance of autoimmunity caused by multiple antigens evoking molecular mimicry during host defense. In addition, results have indicated that recombinant subunit vaccines could directly respond to specific molecular regions conferring protection, indicating that a subunit vaccine could be a safer and targeted choice [26, 27]. Thus, we tested the truncated α-actinin, including the α-actinin forepart (ACT-F) and terminal-part (ACT-T), as subunit immunogens to evaluate their potential in protecting against *T. vaginalis* infection. We found that both ACF-F and ACT-T could confer partial or complete protection in mice against challenge with *T. vaginalis*. To our knowledge, this is the first evidence that demonstrates the effect of an α-actinin subunit as a vaccine candidate against *T. vaginalis* infection in an animal model.

## Methods

### Parasites and animals

The *Trichmonas vaginalis* strain CPOTV21, used in this work, was isolated from an outpatient in the Second Affiliated Hospital of Guangzhou Medical University. It was cryopreserved in liquid nitrogen following axenic cultivation [28]. The parasite was grown in Diamond’s Trypticase-yeast medium [29], supplemented with 10% heat-inactivated fetal bovine serum (Excell, Shanghai, China), 100 U/ml penicillin, 100 μg/ml streptomycin and incubated in 5% CO_2_ at 37 °C. Organisms were subcultured every two days and only the logarithmic-phase parasites were used in this work.

BALB/C mice (about 6 weeks old) and New Zealand white rabbits (about 8 weeks old) were purchased from The Experimental Animal Center of Sun Yat-Sen University (Guangzhou, China). They were all maintained in pathogen free conditions and had free access to food and water according to National Institutes of Health on animal care and the ethical guidelines. Protocols for the use of animals were approved by the Institutional Review Board for Animal Care at Sun Yat-Sen University (#31472058).

### Gene cloning and construction of prokaryotic expression plasmids

Total RNA from *T. vaginalis* was extracted with Trizol reagent (Invitrogen, Carlsbad, USA) following the manufacturer’s instructions. After removal of DNA from RNA with DNase I, the cDNA of *T. vaginalis* was synthesized using oligo (dT) primers and the reverse transcription reaction was conducted by using a primeScript TM RT reagent Kit (Takara, Dalian, China). The α-actinin forepart (ACT-F, 1368 bp, 14–469 aa) and terminal-part (ACT-T, 1146 bp, 462–844 aa,) were separately amplified using the following sets of primers, ACT-F-Fw (with *Kpn I* site in bold): 5′-GAC **GGT ACC** GAG AAG ACC CAG ATC AAG GTT-3′, ACT-F-Rv (with *Sal I* site in bold): 5′-AGT **GTC GAC** GAG GAG GTG CTT GAT GTA TGT-3′, ACT-T-Fw (with *Kpn I* site in bold): 5′-GAC **GGT ACC** ACA TAC ATC AAG CAC CTC CTC-3′, ACT-T-Rv (with *Sal I* site in bold): 5′-AGT **GTC GAC** CTT GCA GTA TTC CTT AGC CTG-3′. PCR amplicons were cloned into expression vector pET-32a (+) and the construction was then transfected into *E. coli* to generate the prokaryotic expression system (BL21-pET32a-TV-α-actinin). Both recombinant cloning vectors and expression vectors were confirmed by double-restriction enzyme digestion and nucleotide sequencing.

### Expression and purification of recombinant proteins

Conditions that predominantly influenced the expression of recombinant α- actinin were optimized through setting a series of gradients, which included initial bacterial density pre-induction, IPTG concentration, induction time and temperature.

The expressed proteins were purified by the His Tag Fusion Protein Purification kit, according to manufacturer’s instruction (Millipore, Billerica, USA). Electroelution was also applied for protein purification as previously described with some changes [30]. Briefly, after SDS-PAGE was finished and the gel was stained with precooled 0.5 mol/l KCl, target bands were minced and washed from white to transparent. Electroelution was implemented by using dialysis bags containing the collected gel in a Tris-glycin buffer (50 mM Tris-base, 50 mM glycin, 0.1% SDS) for 4 h. This was followed by electrophoresis at 100 V constant voltage for another 30 min after exchange of the positive and negative terminals in the electrophoresis apparatus. The purified proteins were then dialyzed in 0.1× PBS overnight and stored at -80 °C after concentration by freeze drying.

### Western blotting

Samples were resolved by SDS-PAGE and then blotted onto nitrocellulose membranes. Antigens were probed by commercial mouse anti-His tag antibodies (1:1000, Abmart, Shanghai, China) or by our own anti-sera (1:2,000) collected from rabbits immunized with purified α- actinin subclones. Horseradish peroxidase (HRP)-labelled anti-mouse IgG or Goat anti-rabbit IgG were used as secondary antibodies (both 1:3,000, Proteintech, Chicago, USA). The color was developed in the dark using DAB for 5–10 min and the reaction was stopped by addition of distilled water.

### Immunization schedule and protection from parasite challenge

New Zealand white rabbits were subcutaneously immunized with purified proteins (ACT-F and ACT-T) for five times (1st, 200 μg emulsified in complete Freund’s adjuvant; 2nd, 200 μg emulsified in incomplete Freund’s adjuvant; 3rd/4th/5th, 100 μg in PBS). This was carried out at one week intervals for acquisition of rabbit anti-sera against the two parts of the TV-α-actinin.

BALB/C mice aged 6 weeks old were randomly divided into seven groups (more than ten in each group), including five experimental groups (ACT-F-high dosage, ACT-F-low dosage, ACT-T-high dosage, ACT-T- Low dosage, Whole-cell antigen) and two control groups (FA-sham and unimmunized). All mice in experimental groups were subcutaneously immunized with a 200 μl volume of vaccines followed by three boosters within a 2-week interval (more details are provided in Table 1). For the FA-sham group, mice were subcutaneously injected with only PBS in Freund’s adjuvant. Ten days after the last immunization, three mice from each group were sacrificed to detect the splenocyte proliferation and cytokines. The remaining animals in each group (number is shown in Fig. 2) were then intraperitoneally challenged with 1 × 10^7^ parasites per mouse. The survival of each individual was recorded daily over a period of a month. The above described immunization experiments were conducted within two experiments each included both experimental and control groups.

**Table 1:** Immunization protocol in experimental and control groups

Groups	Groups (No. of animals)	1st (0w)	2nd (2w)	3rd (4w)	4th (6w)
High dosage (μg)	ACT-F (14)	100	100	100	100
	ACT-T (15)	100	100	100	100
Low dosage (μg)	ACT-F (13)	40	20	20	20
	ACT-T (18)	40	20	20	20
	Whole-cell antigen (13)^a^	40	20	20	20
Control (μl)	FA-sham (14)^b^	200	200	200	200
	Unimmunized (20)^c^	200	200	200	200

^a^ *T. vaginalis* was made into whole-cell antigen by sonication and alternate freeze/thaw treatment (in liquid nitrogen and 37 °C). The whole-cell antigen was quantified by using a Bicinchoninic Acid Kit (BCA). Before vaccination, it was emulsified in Freund’s adjuvant

^b^Each mouse, 100 μl PBS emulsified in 100 μl completed Freund’s adjuvant for the 1st immunization, in incomplete Freund’s adjuvant for 2nd, 3rd and 4th immunizations

^c^Each mouse was injected with only 200 μl sterile PBS using the same procedure in other groups

### Indirect immunofluorescence assays

Animals were bled from the tail one day before *T. vaginalis* challenge. Sera was collected and stored at -80 °C until use. Parasites at logarithmic-phase growth were collected by centrifugation and washed with PBS three times. Half a million cells in 100 μl were added to poly-L-lysine pre-coated slides and air-dried at room temperature for 20 min. After fixation with pre-cooled methanol, the slides were rehydrated with PBS for 20 min and then incubated with anti-sera (1:2,000) collected from mice with or without immunization for one hour. After extensive washes with PBS, another 45 min incubation was undertaken with goat-anti mouse IgG FITC (1:800; Invitrogen) and anti-fade mounting medium with DAPI (50 μg/ml) was conducted. Photos were taken under a fluorescent microscope (Zeiss, Oberkochen, Germany).

### Indirect ELISA for detection of antigen-specific immunoglobulin and subtypes

One day before each immunization, blood samples of the mice with or without immunization were collected and sera were isolated (*n* = 5). To detect the anti-α-actinin specific IgG antibodies and IgG isotypes in the serum samples, indirect ELISA was performed as described in a previous study with some changes [9]. Briefly, microtiter plates were coated with recombinant proteins (2.5 μg/ml, 100 μl/well) for 2 h at 37 °C and blocked with 5% skimmed milk overnight. The plates were then incubated with mouse sera, which were initially diluted at 1:10000 using the half dilution method, to determine the antibody titers at 37 °C for 2 h. To show more detail, mouse sera diluted at 1:320,000 were also incubated with the coated plates for the assessment of total IgG, and diluted at 1:1,000 and 1:200,000 for subtype (IgG1 and IgG2) detection.

The plates were then washed with PBS-T for five times, and the bound antibodies were detected by horseradish peroxidase-conjugated goat anti-mouse IgG, IgG1 and IgG2a (Proteintech, Chicago, USA) diluted at 1:3000. DAB was used to produce the chromogenic reaction and the value was determined using a microplate reader at OD 450 nm (Mutiskan MK3; Thermo Scientific, Waltham, USA). The IgG titers were defined as the highest dilution of sera used when the optical density (OD) values of experimental groups were at least triple that in the negative (unimmunized) sera.

### Splenocyte proliferation

Ten days after the last immunization, spleens were removed aseptically from three mice of each group and passed through nylon sieves to obtain splenocytes. Erythrocytes were removed using NH_4_Cl-Tris lysis solution and the splenocytes were resuspended in complete RPMI-1640 medium supplemented with 10% FBS (Thermo Fisher, Waltham, USA). Cells were then seeded onto 96-well plates at a density of 5 × 10^5^ cells per well and stimulated with antigens dissolved in RPMI-1640 in a final volume of 200 μl per well and cultured at 37 °C with 5% CO_2_. The antigens included recombinant proteins (ACT-F/ACT-T, 10 μg/ml), concanavalin A (Con A, 5 μg/ml, Sigma-Aldrich) or medium alone (negative control). The proliferative activity was measured using a Cell Counting Kit-8 (CCK8, Dojindo laboratories, Kyushu Island, Japan) following the manufacturer’s instructions. Briefly, after 68 h cultivation, 100 μl of the supernatant was removed for cytokine assays. Then 10% CCK8 was added to each well and incubated at 37 °C for 4 h. Thereafter, the absorbance was evaluated at OD 450 nm using a plate reader (Mutiskan MK3; Thermo Fisher). The stimulation index (SI) was calculated as the ratio of the average OD 450 value of wells containing antigen-stimulated cells to the average OD 450 value of wells containing only cells with medium. All assays were performed in triplicate.

### Cytokine assays

For the assessment of cytokines produced by activated splenocytes, the concentration of IL-2, IL-4, IL-6, IFN-γ, IL-17A and IL-10 was determined by using Cytometric Bead Array (CBA) (BD Biosciences, New Jersey, USA). Briefly, according to the manufacturer’s instructions, 50 μl mixture of capturing beads was mixed with 50 μl of supernatant from splenocyte proliferation assay after 68 h cultivation, and then 50 μl of Mouse-PE detection reagent was added for detection. Ten concentration gradients of cytokine standards were used for plotting the standard curve. Mixed samples were incubated for 3 h at room temperature away from light, then washed, centrifuged at 200× *g* for 5 min and subsequently analyzed in the FACScan flow cytometer (BD Biosciences). Each sample was analyzed in duplicate and each group included three mice.

### Statistical analysis

The graphics, including antibody responses, lymphoproliferation assays and cytokine production levels, were made by using GraphPad Prism 5 software. Statistical significance on the comparison of mean values and SEM was assessed by independent sample *t*-test performed using Statistical Package for the Social Sciences (SPSS) version 22.0, IBM. Statistical significance was accepted at *P* < 0.05.

## Results

### Expression of two α-actinin peptides

**Fig. 1:**
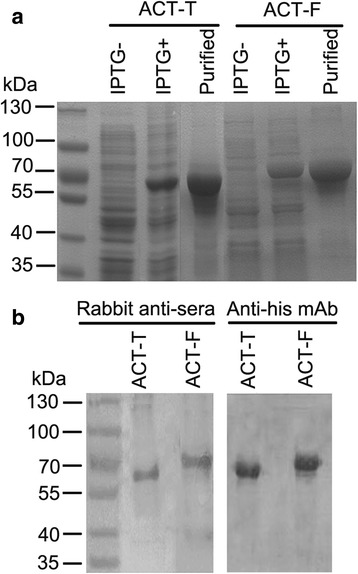
Prokaryotic expression and purification of α-actinin. **a** SDS-PAGE analysis of prokaryotic expression of recombinant α-actinin peptides (ACT-F and ACT-T). IPTG-, non-induced; IPTG+, 0.6 mM IPTG induced for 4 h; purified, after electroelution purification. **b** Western blotting analysis using anti-His tag monoclonal antibody (at 1:1,000 dilution) and sera containing rabbit anti-sera (at 1:2,000 dilution). ACT-F, 70.33 kDa; ACT-T, 61.7 kDa

The coding regions of α-actinin (ACT-F, 14–469 aa and ACT-T, 462–844 aa) were amplified from cDNA and cloned into pET-32a (+) expression vector by insertion at Kpn I/Sal I restriction sites. An attempt was made to keep each subsection of α-actinin an equal length but also containing parts with the predicted highest antigenicity region (amino acid 377 to 382, 409 to 414, and 682 to 687) [21]. After confirmation by double digestion and sequencing, constructs were transfected into BL21 cells. A clear expression of both proteins (70.33 kDa of ACT-F and 61.7 kDa of ACT-T) was achieved by initial induction of the log-phase bacteria (OD 600 nm = 0.8) with IPTG (0.6 mmol/l), at 24 °C for 5 h (Fig. 1a). After Ni-NTA based purification and/or electroelution, we harvested reasonably pure peptides. Western blot by anti-His antibody or anti-sera collected from rabbits immunized with purified proteins confirmed that the peptides were ACT-F and ACT-T (Fig. 1b).

### Protective effect of recombinant antigens

The protective efficacy of the recombinant antigens as potential vaccines against *T. vaginalis* infection was investigated in BALB/C mice. Each mouse received one primary vaccination and three boosters (Table 1), followed by an intraperitoneal injection of 1 × 10^7^ *T. vaginalis* trophozoites.

**Fig. 2:**
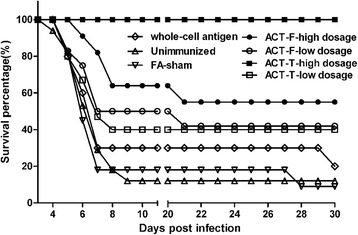
Assessment of protective potency of α-actinin peptide vaccines in mice. BALB/C mice were immunized with ACT-F (high, *n* = 11; low, *n* = 12), ACT-T (high, *n* = 10; low, *n* = 15), whole-cell antigen (*n* = 10), PBS in adjuvant (FA-sham; *n* = 11), unimmunized (*n* = 17) and followed by intraperitoneal challenge with *T. vaginalis* (1 × 10^7^ per mouse) two weeks after last immunization. Mice were monitored daily to obtain the survival percentage

Our data showed that immunization with high dosage of ACT-T gave complete protection to the animals, because 100% of the animals survived for over a month post-infection until they were sacrificed (Fig. 2). In the other four experimental groups, only partial protection (20–55%) was provided by vaccination. For example, the degree of protection was 55% in the ACT-F-high dosage group, 40% in the ACT-T-low dosage group, 42% in the ACT-F-low dosage group and 20% in the whole-cell antigen group. While in the FA-sham and unimmunized groups, most mice (83–89%) were dead within ten days.

### Level of immunoglobulin isotypes in serum samples

**Fig. 3:**
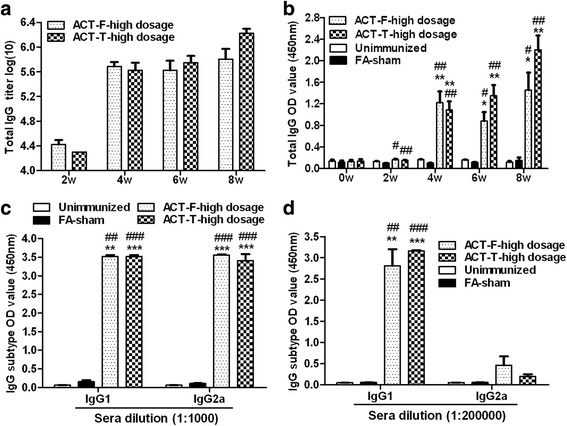
Levels of IgG in the sera of mice immunized with specific antigens. **a** Total IgG titers exhibited, (**b**-**d**) total IgG (at 1:320,000 dilution), IgG subclasses (at 1:1,000 and 1:200,000 dilution) determined by OD450 nm value in the mice sera collected one day before each immunization as indicated. Each bar represents the mean value ± standard error (SEM) (*n* = 5). Significance was accepted by comparison with the unimmunized group (*P < 0.05; **P < 0.01; ***P < 0.001) or FA-sham group (^#^ *P* < 0.05; ^##^ *P* < 0.01; ^###^ *P* < 0.001) using statistical analyses

As the high-dosage immunization groups provided a better protection against *T. vaginalis* infection, the following assessment of vaccine potency only focused on these groups. To determine if a systemic immune response could be stimulated by immunization, a set of mouse sera (5 mice per group) were collected. These were taken at weeks 0, 2, 4, 6 and 8, one day before each scheduled immunization and used for the detection of the α-actinin specific immunoglobulin IgG. As indicated in Fig. 3a, both the recombinant proteins (ACT-F and ACT-T) could induce a high titer of IgG in BALB/C mice.

The IgG titer could be detected at two weeks after the primary immunization and reached a peak only if mice received another booster injection (at the 4th week) (Fig. 3a). This indicated that a total of two vaccinations may be enough for protection. While an OD 450 nm value of serum IgG at 1:320000 dilution gradually increased with each vaccination (Fig. 3b). As expected, sera collected from the mice in FA-sham and unimmunized groups were negative, showed no specific antibody titer and displayed a background level of the OD 450 value even at a 1:500 dilution (Fig. 3b).

The OD 450 values of the ACT-F-high dosage and ACT-T-high dosage specific IgG1 (Fig. 3c, d) in the vaccinated mice were significantly higher than that in the unimmunized (ACT-F: *t*_(4)_ = -7.105, *P* = 0.002; ACT-T: *t*_(4.003)_ = -146.210, *P* < 0.0001) and sham FA-sham groups (ACT-F: *t*_(4.001)_ = -7.087, *P* = 0.002; ACT-T: *t*_(4.197)_ = -144.152, *P* < 0.0001), which were seen only at a background level. The high levels of IgG1 compared with IgG2a (ACT-F: *t*_(8)_ = 5.321, *P* = 0.001; ACT-T: *t*_(8)_ = 50.741, *P* < 0.0001) in each experimental group indicated that the humoral immunization elicited by these two peptides was a mix of Th1 and Th2 responses but predominantly biased towards the Th2 response (Fig. 3c, d).

### Anti-sera generated recognized α-actinin

**Fig. 4:**
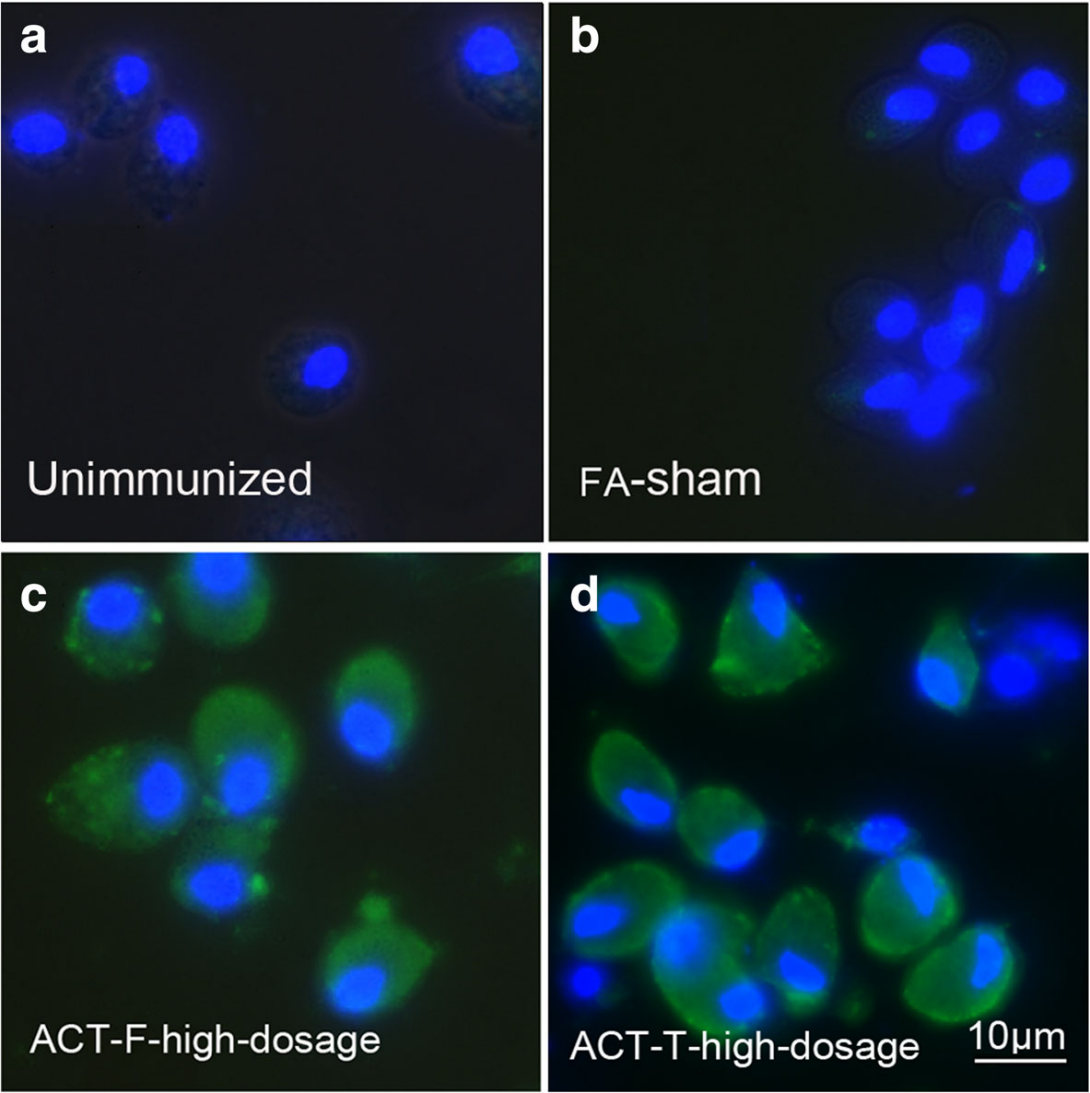
Native α-actinin in *T. vaginalis* was recognized by serum from immunized mice*.* Immunofluorescence of native parasite α-actinin detected with mouse sera (at 1:2,000 dilution) from different groups: **a** *T. vaginalis* were incubated with pre-immunized mouse serum; **b** *T. vaginalis* were incubated with serum from FA-sham immunized mice; **c** *T. vaginalis* were incubated with serum from high dosage ACT-F immunized mice; **d** *T. vaginalis* were incubated with serum from high dosage ACT-T immunized mice serum. Goat-anti mouse IgG FITC (at 1:800 dilution) was used as secondary antibody. *T. vaginalis* α-actinin was labeled with FITC (*in green*) and DNA was stained with DAPI (*in blue*). *Scale-bar*: 10 μm

Immunofluorescence assays were performed to assess whether anti-sera against α-actinin could recognize the native protein in *T. vaginalis* trophozoites. Diluted immune sera (1:1,000) and pre-immune sera from mice were incubated with fixed parasites and were further reacted with goat-anti mouse IgG FITC and DAPI. According to other research, intracellular α-actinin is located throughout the cytoplasm [21]. Our study showed that, when probed with immune sera from the two protein-immunized groups, the native α-actinin could be detected as being diffusely distributed in the cytoplasm (Fig. 4c, d). However, it was not detected in control groups treated with the pre-immune or FA-sham sera (Fig. 4a, b). This recognition may support the possibility that specific antibodies could also recognize α-actinin of *T. vaginalis* in vivo to further mediate antibody dependent cytotoxicity.

### Splenocyte proliferation assay

**Fig. 5:**
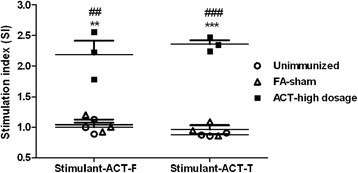
Proliferation analysis of splenocytes from immunized mice in vitro. Splenocytes from ACT-F- high dosage, ACT-T- high dosage, FA-sham (PBS in adjuvant) and unimmunized groups were stimulated with ACT-F or ACT-T (10 μg/ml) for 68 h in vitro. Proliferation was determined using CCK8 cell counting kits. The stimulation index (SI) is calculated as the ratio of the proliferation of stimulated cells to non-stimulated cells in the same group. The data are the mean SI ± SEM from three individual mice from each group with three repeats (*n* = 3). Significance was accepted by comparison with the unimmunized (*P < 0.05; **P < 0.01; ***P < 0.001) and FA-sham groups (^#^ *P* < 0.05; ^##^ *P* < 0.01; ^###^ *P* < 0.001) using statistical analyses

To further assess the immune responses, splenocytes were removed from mice in each group and proliferation was measured upon stimulation with corresponding ACT-F and ACT-T proteins (10 μg/ml) in response to immunization. As shown in Fig. 5, almost no increase in proliferation (approximate stimulation index, SI = 1.0) was detected in splenocytes from mice in unimmunized and FA-sham groups after stimulation with either ACT-F or ACT-T. While for splenocytes from mice immunized with high dosages of ACT-F or ACT-T, significant increases in stimulation index (SI) were observed with re-exposure to either ACT-F (ACT-F *vs* FA-sham: *t*_(4)_ = -4.793, *P* = 0.009) or ACT-T (ACT-T *vs* FA-sham: *t*_(4)_ = -14.897, *P* < 0.0001). The proliferation of lymphocytes in samples indicated that immune memory responses were present and might play a protective function against parasites with the same antigens. In addition, after treatment with ConA (10 μg/ml, positive control), splenocytes from mice in all groups proliferated to comparable levels as those being stimulated with ACT-F and ACT-T (SI = 1.67–2.34).

### Cytokine expression

**Fig. 6:**
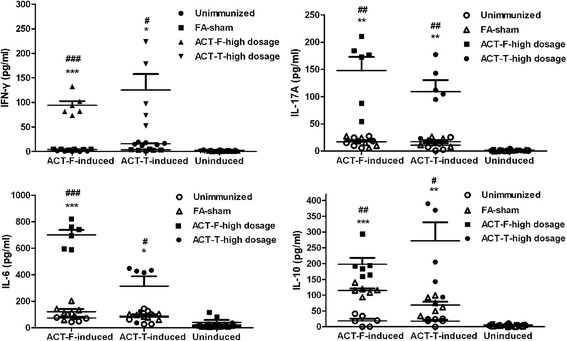
Cytokine secretion of splenocytes from immunized mice. Cytokines in supernatants were assayed in duplicate using CBA Kits (*n* = 3). The data are shown as mean value ± standard error (SEM) for antigen treatment. Significance was calculated by comparison with the unimmunized group (*P < 0.05; **P < 0.01; ***P < 0.001) or the FA-sham group (^#^ *P* < 0.05; ^##^ *P* < 0.01; ^###^ *P* < 0.001) using statistical analyses

After the splenocyte proliferation assays were carried out, cytokine secretion in the supernatants was tested. As indicated in Fig. 6, in vitro treatment of splenocytes from the high dosage ACT-T vaccinated mice with ACT-T triggered significantly higher amounts of pro-inflammatory cytokines such as IFN-γ (36.7 fold; FA-sham, 3.41 pg/ml; ACT-T, 125 pg/ml; *t*_(4)_ = -3.734, *P* = 0.02), IL-17A (6.34 fold; FA-sham, 17.2 pg/ml; ACT-T, 109 pg/ml; *t*_(10)_ = -4.31, *P* = 0.002) and IL-6 (3.57 fold; FA-sham, 88.1 pg/ml; ACT-T, 314 pg/ml; *t*_(5.084)_ = -3.011, *P* = 0.029). While mice immunized with the high dosage of ACT-F, splenocytes pretreated with ACT-F could also secrete significantly high levels of IFN-γ (20.3 fold; FA-sham, 4.63 pg/ml; ACT-F, 94.2 pg/ml; *t*_(5.015)_ = -10.289, *P* < 0.0001), IL-17A (8.81 fold; FA-sham, 16.8 pg/ml; ACT-F, 148 pg/ml; *t*_(5.179)_ = -5.157, *P* = 0.003) and IL-6 (5.74 fold; FA-sham, 122 pg/ml; ACT-F, 700 pg/ml; *t*_(10)_ = -13.46, *P* < 0.0001) when compared with unimmunized and FA-sham groups, which only produced background levels of these cytokines (Fig. 6). As a marker for the Th2 response, IL-10 detection was increased significantly in the mice vaccinated with high dosage of ACT-T (3.94 fold; FA-sham, 69.0 pg/ml; ACT-T, 272 pg/ml; *t*_(5.309)_ = -3.428, *P* = 0.017) and high dosage of ACT-F (1.71 fold; FA-sham, 115 pg/ml; ACT-F, 197 pg/ml; *t*_(10)_ = -3.923, *P* = 0.003) respectively. This might be caused by the requirement for an immune Th1/Th2 balance in vivo for host self-protection [31].

In response to ConA, the positive control, high levels of the above-mentioned cytokines were detected in the splenocytes isolated from animals in all groups (data not shown). Interestingly, however, as common markers of Th1 (IL-2) and Th2 (IL-4) immune responses, only barely detectable levels of IL-2 (4.87–21.3 pg/ml) and IL-4 (4.20–21.3 pg/ml) were seen in cells from all groups after restimulation in vitro.

## Discussion

For prevention and control of infectious diseases, vaccines are usually considered the most cost-efficient tools to block the transmission of pathogens [32]. However, the search for candidate antigens is one of the most difficult steps in the process. Several reports have suggested α-actinin of *T. vaginalis* as a candidate for the development of effective vaccines against this parasite [7, 16, 24].

Alpha-actinin, both native and recombinant, can be recognized by sera from women who have been exposed to *T. vaginalis*. It has been proved to be the most common immunogen with high immunogenicity [9, 24, 33], an essential requirement as a vaccine candidate [32]. Additionally, there is a very low similarity in amino acid sequences of the epitopes between *T. vaginalis* α-actinin and the corresponding human actinin homolog [21, 34]. This could avoid the possibility of autoimmunity caused by epitope similarity in humans. Most importantly, as a structural protein, α-actinin is constantly expressed during the entire life-cycle of *T. vaginalis* and is present in all isolates despite geographic variation [24]. Therefore, these broadly positive characteristics make the development of α-actinin as a candidate vaccine inspiring and meaningful.

For the assessment of a vaccine candidate, protective efficacy in vivo is one of the most important parameters [32]. As a commonly used method in parasitology for measuring protective efficacy, the survival rate of vaccinated animals after challenge with pathogens (in this case, *T. vaginalis*) is a direct parameter. Our results showed that immunization with a high dose of ACT-T gave complete protection with 100% animals immunized surviving for over a month post-infection until the experiment was terminated (Fig. 2). Comparatively, low-dosage ACT-T or ACT-F also provided a protective effect (40% or 42%, respectively) that was still higher than those immunized with whole-cell antigen (20%). These results indicated that these recombinant proteins showed higher protective potency than whole-cell antigen in our mouse model. The results also clearly demonstrate that both ACT-F and ACT-T, but particularly the high dosage ACT-T, could significantly protect mice from the challenge by *T. vaginali*s. This strongly supports the suggestion that α-actinin could be a candidate for the development of an effective vaccine against *T. vaginalis* [7, 16, 24].

Previously, there have been some attempts to develop vaccines against *T. vaginalis* [17, 18]. Of which, two studies relied on a whole-cell *T. vaginalis* vaccine given by subcutaneous immunization and vaginal challenge of *T. vaginalis* in mice*.* Although 72–100% protection was found, it could be questioned as to whether their infection was initially successful. Remarkably, in their detection at one month after *T. vaginalis* infection, the parasite was not detected in 39–53% of the mice in their unimmunized group [17, 18]. This means that nearly half of the mice could, without any vaccination, either eliminate *T. vaginalis* in their vaginas directly and/or have failed to get a successful infection. Their results indicated that the model of vaginal inoculation with *T. vaginalis,* even with pretreatment of *Lactobacillus acidophilus* and estradiol valerate in the mouse vagina*,* may provide inconsistent infection rates (in this case varying from 47–61%) [17, 18]. Additionally, in their studies, parasites were not recovered in 43% of the mice in the FA-sham group or 80% of the mice in the Alum-sham group. It seemed that the adjuvants, especially Alum, as opposed to the whole-cell *T. vaginalis*, mainly played the protective function. It is obvious that the mouse model, using vaginal infection, can’t mimic and be considered a suitable model to evaluate the effect of vaccines against *T. vaginalis* infection. In fact, this model not only provides unreliable results compared to our current study but reliance on it also limits the progress of related research.

In contrast, in our current study, almost 90% of the mice died in the Freund’s adjuvant-sham (FA-sham) and unimmunized groups within 10 days after intraperitoneal challenge with 1 × 10^7^ *T. vaginalis*. This clearly indicated a high and guaranteed initial infection in our intraperitoneal mouse model. Intraperitoneal inoculation of the parasite in mice is a common model for detection of virulence of *T. vaginalis* isolates or for screening of trichomonacidal compounds [35, 36]. In our understanding, this model also mimics a natural migration of *T. vaginalis* to the peritoneal cavity causing ascites, which has been reported in patients [37, 38]. Importantly, the intraperitoneal infection model has also been used in other relevant studies for vaccine candidate assessment [39].

Therefore, we consider that it is a reliable model for vaccine development against *T. vaginalis* with the following advantages: (i) Clinical signs in the animal, such as curling up, lack of vitality, decreased appetite, piloerection in fur, sloth and weight loss, could be clearly observed. Almost all mice without vaccination with α-actinin died within 10 days post-inoculation of *T. vaginalis* strain CPOTV21, which makes it easier to evaluate the protection of the potential proteins. (ii) Several years experience in our laboratory has demonstrated that the mouse peritoneal cavity is an ideal place for *T. vaginalis* growth compared with the traditional mouse vagina. (iii) The intraperitoneal inoculation model was convenient to operate and guaranteed infection, while the intravaginal model is time consuming and involves a complicated pretreatment process. For example, it requires pretreatment of the mouse vagina with estrogen and *L. acidophilus* to establish a sustainable infection and it requires rinses to collect the living parasites before each examination of infection [40]. Despite the pretreatment, infection ratios of 47 to 61% in the intravaginal model were still low and therefore it should not be considered a suitable animal model for vaccine development and anti-trichomonas compound screening.

To further evaluate the vaccine potency of our proteins, important parameters were assessed including measuring the humoral immune response status, T lymphocyte proliferation capability and cellular immune response status. We found that both ACT-F and ACT-T could induce a mixed Th1/Th2 response with a high level of specific IgG but with Th2-bias isotypes (IgG1 > IgG2) in mice (Fig. 3c). This indicates that they are highly immunogenic proteins. This is in accordance with the results from other laboratories [17–19], from which a prominent increase of IgG1 was also detected. However, although *T. vaginalis* antibodies were frequently detected in patients, protection by these immunoglobulins was not found [25, 41, 42]. Therefore, to our knowledge, the exact function of immunoglobulins in host protection against *T. vaginalis* remains unclear. Based on our results, the high antibody titers and protection found in the mice immunized with high dosage of ACT-T and the lesser effect with low dosage of ACT-T indicate that the protection against *T. vaginalis* infection may possibly link with humoral responses, at least in part.

There is no doubt that besides the humoral responses, the cellular immune response is also an important index for assessing the immunogenicity of vaccine candidates. For this purpose, the level of cytokines in the supernatant of lymphocyte cultures from spleen following immunization and re-stimulation in vitro is usually one of the detectable marker systems*.* Cytokines released by lymphocytes, especially the Th1-type (e.g. IFN-γ) play an important function in host protection against many pathogen infections including *T. vaginalis* [43]. Our results also demonstrated that significantly higher levels of IFN-γ were detected in the animals vaccinated with ACF-F or ACT-T than those from the controls which were in accord with the protection rate seen. In fact, IFN-γ has been proved to increase macrophage-mediated cytotoxity against *T. vaginalis* [44, 45]. Of which the monocyte-macrophage lineage has been identified as an important lineage involved in *T. vaginalis* killing in vivo [46]. In other studies, IFN-γ has also been considered to play a role in the elimination or suppression of proliferation of *T. vaginalis* [43–45] by stimulation of nitric oxide production as one of the effectors [44, 45].

IL-17, another crucial proinflammatory factor, was also found to be significantly increased in splenocytes from the animals immunized with ACF-F/T in our work. Although the role of IL-17 is poorly understood during *T. vaginalis* infection, data from patients infected with *T. vaginalis* [47] and our work indicated that IL-17 could be involved in protection. As a matter of fact, IL-17 has been shown to play important roles in host responses against pathogens like *Entamoeba histolytica* [48] and some bacterial infections [49, 50]. These IL-17-mediated protective effects may function by a direct or indirect regulation of Th1 responses, dendritic cells and neutrophils [48–51]. Of them, neutrophils have been shown to be a major host protector during *T. vaginalis* infection either through a complement-mediated and IgG-enhanced pathway or by the myeloperoxidase and superoxide anion cytotoxity pathways [52–54]. IL-17 is a key cytokine for the activation, recruitment, and migration of neutrophils to the inflammation sites [55, 56]. It is likely, therefore, that the observed increase in IL-17 in the animals vaccinated with ACT is likely to be involved in a protective role, through the neutrophil-dependent mechanism, in trichomoniasis. Obviously, further research is required before we can better understand the mechanism of action of this cytokine in *T. vaginalis* infection.

## Conclusions

In summary, the present study demonstrated that a high dose of recombinant *T. vaginalis* α-actinin fragments, derived from ACT-F and ACT-T, could induce a mixed (Th1/Th2) humoral and cellular immune response and provide 48% and 100% protection in mice through multifaceted mechanisms. These results strongly support the view that α-actinins, which are characterized by high immunogenicity, high antigen specificity and well conserved immunogenic epitopes, are strong potential vaccine candidates against *T. vaginalis* infection.

## Abbreviations

ACT-F: α-actinin forepart
ACT-T: α-actinin terminal-part
CBA: Cytometric Bead Array
CCK8: Cell counting kit-8
IELISA: Indirect enzyme-linked immunosorbent assay
IPTG: Isopropyl-Beta-D-hiogalactopyranoside
TV: *Trichomonas vaginalis*
